# Treatment responses to Azithromycin and Ciprofloxacin in uncomplicated *Salmonella* Typhi infection: A comparison of Clinical and Microbiological Data from a Controlled Human Infection Model

**DOI:** 10.1371/journal.pntd.0007955

**Published:** 2019-12-26

**Authors:** Celina Jin, Malick M. Gibani, Shaun H. Pennington, Xinxue Liu, Alison Ardrey, Ghaith Aljayyoussi, Maria Moore, Brian Angus, Christopher M. Parry, Giancarlo A. Biagini, Nicholas A. Feasey, Andrew J. Pollard

**Affiliations:** 1 Oxford Vaccine Group, Department of Paediatrics, University of Oxford, and NIHR Oxford Biomedical Research Centre, Oxford, United Kingdom; 2 Department of Infectious Diseases, Imperial College London, London, United Kingdom; 3 Research Centre for Drugs and Diagnostics, Liverpool School of Tropical Medicine, Liverpool, United Kingdom; 4 Nuffield Department of Medicine, University of Oxford, Oxford, United Kingdom; 5 Department of Clinical Sciences, Liverpool School of Tropical Medicine, Liverpool, United Kingdom; 6 Institute of Infection and Global Health, University of Liverpool, Liverpool, United Kingdom; 7 School of Tropical Medicine and Global Health, Nagsaki University, Nagasaki, Japan; 8 Malawi Liverpool Wellcome Trust Clinical research Programme, Blantyre, Malawi; Johns Hopkins Bloomberg School of Public Health, UNITED STATES

## Abstract

**Background:**

The treatment of enteric fever is complicated by the emergence of antimicrobial resistant *Salmonella* Typhi. Azithromycin is commonly used for first-line treatment of uncomplicated enteric fever, but the response to treatment may be sub-optimal in some patient groups when compared with fluoroquinolones.

**Methods:**

We performed an analysis of responses to treatment with azithromycin (500mg once-daily, 14 days) or ciprofloxacin (500mg twice-daily, 14 days) in healthy UK volunteers (18–60 years) enrolled into two *Salmonella* controlled human infection studies. Study A was a single-centre, open-label, randomised trial. Participants were randomised 1:1 to receive open-label oral ciprofloxacin or azithromycin, stratified by vaccine group (Vi-polysaccharide, Vi-conjugate or control Men-ACWY vaccine). Study B was an observational challenge/re-challenge study, where participants were randomised to challenge with *Salmonella* Typhi or *Salmonella* Paratyphi A. Outcome measures included fever clearance time, blood-culture clearance time and a composite measure of prolonged treatment response (persistent fever ≥38.0°C for ≥72 hours, persistently positive *S*. Typhi blood cultures for ≥72 hours, or change in antibiotic treatment). Both trials are registered with ClinicalTrials.gov (NCT02324751 and NCT02192008).

**Findings:**

In 81 participants diagnosed with *S*. Typhi in two studies, treatment with azithromycin was associated with prolonged bacteraemia (median 90.8 hours [95% CI: 65.9–93.8] vs. 20.1 hours [95% CI: 7.8–24.3], p<0.001) and prolonged fever clearance times <37.5°C (hazard ratio 2.4 [95%CI: 1.2–5.0]; p = 0.02). Results were consistent when studies were analysed independently and in a sub-group of participants with no history of vaccination or previous challenge. A prolonged treatment response was observed significantly more frequently in the azithromycin group (28/52 [54.9%]) compared with the ciprofloxacin group (1/29 [3.5%]; p<0.001). In participants treated with azithromycin, observed systemic plasma concentrations of azithromycin did not exceed the minimum inhibitory concentration (MIC), whilst predicted intracellular concentrations did exceed the MIC. In participants treated with ciprofloxacin, the observed systemic plasma concentrations and predicted intracellular concentrations of ciprofloxacin exceeded the MIC.

**Interpretation:**

Azithromycin at a dose of 500mg daily is an effective treatment for fully sensitive strains of *S*. Typhi but is associated with delayed treatment response and prolonged bacteraemia when compared with ciprofloxacin within the context of a human challenge model. Whilst the cellular accumulation of azithromycin is predicted to be sufficient to treat intracellular *S*. Typhi, systemic exposure may be sub-optimal for the elimination of extracellular circulating *S*. Typhi. In an era of increasing antimicrobial resistance, further studies are required to define appropriate azithromycin dosing regimens for enteric fever and to assess novel treatment strategies, including combination therapies.

**Trial registration:**

ClinicalTrials.gov (NCT02324751 and NCT02192008).

## Author summary

Typhoid fever remains a major global health problem in low-and middle-income countries. The treatment of typhoid fever is complicated by the emergence of widespread antibiotic resistance. Azithromycin is presently one of the few oral antibiotic options that can be reliably used for typhoid treatment, although concerns persist regarding variations in response to treatment and emerging resistance. We used a *Salmonella* human challenge model to better understand response to treatment with azithromycin, as compared with ciprofloxacin. We demonstrate that azithromycin effectively treated typhoid fever, within the context of the model, however longer durations of bacteraemia and fever clearance times (defined as >37.5°C) were observed when compared with ciprofloxacin treatment. We studied the pharmacokinetic properties of azithromycin and ciprofloxacin within the model to better characterise appropriate dosing strategies. We conclude that oral azithromycin is an effective treatment option for uncomplicated enteric fever in the outpatient setting and should be used in high-burden countries where fluoroquinolone-resistance is common. This study illustrates the application of human challenge models to study antibiotic treatments for typhoid fever. Further studies are required to assess novel treatment strategies, including appropriate azithromycin dosing regimens and combination therapies.

## Introduction

Typhoid fever remains a major global-health concern and is estimated to be responsible for an estimated 10.9 million cases, and approximately 117,000 deaths, annually [1]. The mortality rate of enteric fever in the pre-antibiotic era was estimated to be 10–30% [2]. However, the availability of effective antimicrobial therapy over the past 70 years has reduced the overall mortality rate to <1% [3]. The recent emergence and global dissemination of multi-drug resistant and fluoroquinolone resistant strains of *S*. Typhi and Paratyphi has limited effective treatment options for enteric fever [4]. The World Health Organisation (WHO) has recently listed fluoroquinolone-resistant *Salmonella* as a “priority pathogen”, identified as one of 12 families of bacteria thought to pose the greatest risk to human health through rising antimicrobial resistance [5].

Treatment options for enteric fever–particularly in the outpatient setting–are now severely limited. Third generation cephalosporins are commonly used in the empirical treatment of enteric fever and are a valuable treatment option in the setting of MDR and fluoroquinolone resistant isolates [6]. Several recent reports have described the emergence of extended spectrum beta-lactamase producing *S*. Typhi [7,8]—including a strain of extensively drug resistant (XDR) *S*. Typhi genotype 4.3.1 (H58) responsible for a large typhoid outbreak in Pakistan [9,10]. Strains resistant to third-generation cephalosporins may require treatment with carbapenems, which are either unavailable or prohibitively expensive in resource limited settings [11].

Azithromycin is an azalide antimicrobial widely used for the empirical treatment of uncomplicated enteric fever, benefitting from once daily oral dosing and good tissue penetration. It has excellent *in vitro* activity, being concentrated within phagocytic cells and achieving intracellular concentrations of up to 200 times greater than serum [12]. Several randomised controlled trials have demonstrated the efficacy of azithromycin in adults and children when compared with fluoroquinolones, cephalosporins and chloramphenicol—including in the treatment of fluoroquinolone intermediate or resistant strains [13–20]. Resistance to azithromycin amongst circulating *S*. Typhi strains is uncommon but appears to be an emerging problem. A recent study of >1000 isolates collected between 2013 to 2016 in Dhaka, Bangladesh identified 12 azithromycin-resistant *S*. Typhi strains and one *S*. Paratyphi strain [21]. Azithromycin can be associated with fever-clearance times averaging 4–5 days [13,16–18]; prolonged bacteraemia of up to 72–96 hours post treatment [13,19] and treatment failures [18,22]. Sub-optimal treatment responses are associated with increased morbidity, as well as having potentially harmful effects through prolonged treatment courses, interrupted treatment regimens (prompted by escalation or switching antibiotics) and increased healthcare burden.

Controlled human infection (CHI) models have previously been applied to study antibiotic therapy following *S*. Typhi challenge [23]. Such studies offer the advantage of accurately recording clinical treatment responses in a closely monitored experimental setting with daily collection of culture samples to accurately determine the dynamics of bacteraemia. In light of the increasingly limited treatment options for enteric fever, we sought to compare treatment responses to azithromycin and ciprofloxacin in healthy volunteers challenged with a fully antibiotic susceptible strain of *S*. Typhi as part of a programme of controlled human infection studies.

## Methods

### Ethics statement

Study protocols were approved by the sponsor (University of Oxford), the South-Central Oxford A Ethics Committee (14/SC/1427, 14/SC/1204), and the Medicines and Healthcare Products Regulatory Agency (Eudract 2014-002978-36). Both studies are registered with ClinicalTrials.gov (NCT02324751, NCT02192008).

### Study design

We performed a secondary analysis of two *S*. Typhi controlled human infection studies (Studies A and B) comparing treatment responses to oral azithromycin and ciprofloxacin in participants diagnosed with uncomplicated typhoid fever. Both studies were conducted at the Centre for Clinical Vaccinology and Tropical Medicine, Oxford, United Kingdom from March 2015 to August 2017.

### Study participants

Participants from Study A were blinded, randomised and vaccinated with a single dose of Vi-tetanus toxoid conjugate (Vi-TT; Typbar-TCV, Bharat Biotech, Hyderabad, India), Vi-polysaccharide (Vi-PS; TYPHIM Vi, Sanofi Pasteur, Lyon, France) or control meningococcal ACWY-CRM conjugate vaccine (MENVEO, GlaxoSmithKline), one month prior to *S*. Typhi oral challenge (10^4^ CFUs of *S*. Typhi Quailes strain administered with sodium bicarbonate) [24]. Pre-treatment with sodium bicarbonate allowed increased passage of *S*. Typhi through the gastric acid barrier to achieve an attack rate of 60–75% in control vaccinated participants [24,25]. At the time of enrolment, participants were also randomised to receive 14 days of open-label treatment with either azithromycin 500mg daily or ciprofloxacin 500mg twice daily. Antibiotic randomisation was stratified according to vaccine group. Randomisation to vaccine group and antibiotic treatment was implemented using the computerised randomisation software Sortition (Nuffield Department of Primary Care, Clinical Trials Unit, University of Oxford)

Study B was an observational challenge-re-challenge study. Naïve participants and those with previous exposure to *S*. Typhi or *S*. Paratyphi in earlier challenge studies[24,26–28], were randomised to challenge/re-challenge with either 10^4^ CFUs of *S*. Typhi (Quailes strain) or 10^3^ CFUs of *S*. Paratyphi A (NVGH308). Antibiotic allocation in Study B was not randomised. Between March 2015 to October 2016, the protocol specified that azithromycin 500mg daily was to be used as first-line treatment for all participants. Following the availability of preliminary results from Study A–and under the guidance of the Data Safety Monitoring Committee–the protocol was amended such that first-line treatment was changed to ciprofloxacin 500mg twice daily.

Written informed consent was obtained from all volunteers before enrolment. Inclusion and exclusion criteria have previously been described [24,26,27].

### Study procedures

Study procedures, including preparation and administration of the challenge agent (10^4^ CFUs of *S*. Typhi Quailes strain), diagnostic criteria for typhoid fever, and clinical assessment were identical between studies, and were carried out as previously described [24,26,27]. Briefly, participants were reviewed daily in an outpatient setting for two-weeks following oral challenge. Blood cultures (BD BACTEC Automated Blood Culture System, 10ml) and oral temperatures were collected during clinical visits. Solicited symptoms of typhoid fever and twice-daily self-measured oral temperatures were recorded by participants on an electronic diary for 21 days (inclusive of the two-week challenge period). Antibiotic treatment was commenced if participants were diagnosed with typhoid fever, based on pre-specified composite criteria (*S*. Typhi bacteraemia and/or persistent fever ≥38°C for ≥12 hours) or at the end of the two-week challenge period if participants remained undiagnosed. Diagnosed participants attended between four to seven additional daily visits after commencing antibiotics to assess treatment response. Antibiotic allocation was unblinded. In instances where treatment was changed, the antibiotic switch was performed at the discretion of the treating clinician and Chief Investigators either for adverse reactions, possibly related to antibiotic allocation (e.g. suspected drug-induced liver injury) or for suspected treatment failures or delayed treatment response (e.g. persistent fever or unanticipated symptom severity for the stage of treatment).

### Challenge strain

Typhoid challenge was performed using *S*. Typhi Quailes strain (genotype 3.1.0)[29], which was isolated from the gallbladder of a typhoid carrier in 1958. Challenge was performed using frozen cell banks manufactured to Good Manufacturing Practices (GMP) guidelines and stored at -80°C as previously described [24,26,27]. Challenge strain stocks were fully sensitive to ciprofloxacin and azithromycin as assessed using disc diffusion (zone of inhibition to ciprofloxacin 37mm, azithromycin 24mm). The MIC of *S*. Typhi Quailes strain to ciprofloxacin was 0.016μg/ml (sensitive) and the azithromycin MIC was 6μg/ml (sensitive), as determined by ETEST (BioMeriux).

### Outcome measures

The primary objective of the study was to compare the effect of ciprofloxacin with azithromycin on the time to bacteraemia clearance and fever clearance in individuals diagnosed with typhoid fever. Time to bacteraemia clearance was defined as time from initiation of antibiotics to time of collection of first persistently negative blood culture for *S*. Typhi. Only individuals who were bacteraemic at the time of initiation of antibiotics were included in the analysis of blood culture duration. Fever clearance time (FCT38) was defined as time from antibiotic commencement (or time of fever ≥38.0°C onset if occurring after commencing antibiotics) to time of first persistent temperature <38.0°C after starting of antibiotic treatment. Individuals who cleared their fever before antibiotic initiation were excluded from the fever clearance analysis.

Secondary outcomes included comparison of the effect of antibiotic treatment on FCT <37.5°C; time to symptom resolution (defined as reporting no solicited symptoms of typhoid fever); time to cessation of stool shedding (measured as time from commencement of antibiotics to time of first negative *S*. Typhi stool culture if this occurred after starting antibiotics), and prolonged treatment response (defined as persistent *S*. Typhi bacteraemia and/or persistent fever ≥38°C for ≥72 hours after commencing antibiotics and/or change in antibiotic treatment due to adverse reactions or clinical concerns).

### Laboratory assays

Blood and stool culture samples were collected at 12 hours after challenge and daily thereafter until 96 hours post initiation of treatment [24]. Samples were processed by the local hospital’s accredited laboratories as previously described [30]. Additional pharmacokinetic studies (measurement of ciprofloxacin or azithromycin drug levels in plasma) and comparison of liver enzyme derangement were carried out in Study A participants. Plasma samples from Study A participants were collected at the time of diagnosis (prior to commencing antibiotics) and 12, 24, 48, 72- and 96-hours post-diagnosis for pharmacokinetic studies. Quantitative blood culture was performed using 10ml ISOLATOR tubes (Abbot, UK) as previously described [24].

### Quantification of azithromycin and ciprofloxacin from plasma

Plasma samples (100μL for azithromycin and 50μL for ciprofloxacin) were prepared in 300μL of acetonitrile containing either 20ng/mL azithromycin-D3 (azithromycin) or ciprofloxacin-D8 (ciprofloxacin). 25μL of each sample was loaded onto a Hypersil C8 GOLD high-performance liquid chromatography (HPLC) column (1.9 μm, 100 mm × 2.1 mm; Thermo Scientific). Separation was achieved using a rapid stepwise gradient. Mobile phase A consisted of 0.5% formic acid in water and mobile phase B consisted of acetonitrile (ACN). Initial conditions consisted of 90% mobile phase A, from 0 to 0.2 mins increasing in organic content to 90% mobile phase B from 0.2 to 0.26 and held over 3.74 minutes (azithromycin) or 1.74 minutes (ciprofloxacin). The column was then equilibrated to the initial conditions over 6 minutes (azithromycin) or 4 minutes (ciprofloxacin). The triple quadrupole mass spectrometer (TSQ Quantum Access; Thermo Electron Corporation) was operated in positive ionization mode, and detection and quantification performed using selective reaction monitoring. Assays were validated over calibration ranges of 10–2,560ng/mL (azithromycin) or 10–2,000ng/mL (ciprofloxacin). The lower limit of quantification (10ng/ml) was set as the lowest point on the standard curve with a coefficient of variation (CV) less than 10%. Inter-day precision, based on CV of quality controls, was between 1.8%-7.5% (azithromycin) and 1.7%-7.3% (ciprofloxacin). All quality controls readings at low, medium and high levels were consistent and reproducible.

### Pharmacokinetic (PK) Monte-Carlo simulations

To simulate predicted azithromycin plasma and intracellular pharmacokinetic profiles, we applied a model previously described by Sampson and colleagues [31] and replicated using the simulator tool in Pmetrics [32]. For each dosing scenario, we simulated the PK profile of azithromycin in plasma and intracellularly (mononuclear cells) in 1,000 patients. The PK profile of ciprofloxacin in systemic plasma was simulated in 1,000 patients for each dosing scenario using the model reported by Sanchez Navarro and colleagues [33] and was again replicated using the simulator tool of Pmetrics.

### Statistical analysis

Intention-to-treat analyses were used to assess outcome measures for Study A (antibiotic allocated based on randomisation) and Study B (first antibiotic treatment used). Primary and secondary outcomes were summarised using the Kaplan-Meier method and antibiotic group comparisons were performed using log-rank tests. As no further blood cultures were scheduled for collection after 96 hours post-typhoid diagnosis, the analysis for duration of bacteraemia were censored at 96 hours after treatment initiation. Symptom and temperature data were censored 21 days after challenge, or at the time of last symptom reporting if participants were lost to follow-up.

Adjusted analyses of time to event variables were conducted using the Cox proportional hazards model. The following covariates were included in the model: antibiotic allocation, vaccine assignment, naïve versus previous *S*. Typhi or *S*. Paratyphi challenge, time to antibiotic commencement from time of challenge, and study enrolment (i.e. Study A versus Study B). In addition, sensitivity analyses were carried out with further adjustment for blood quantification results (i.e. number of *S*. Typhi bacteria CFUs isolated from blood at the time of diagnosis) to assess the effect of *S*. Typhi bacterial burden on infection resolution. Subgroup analysis was carried out in participants with no history of vaccination and those with no previous challenge.

P values less than 0.05 were considered significant for the main comparisons (FCT and bacteraemia clearance). As a large number of subsidiary analyses were undertaken, a more stringent criteria of p<0.001 was used to judge the significance of secondary outcomes. All analyses were performed using Stata v15.1 (StataCorp LP, College Station, TX, USA).

## Results

**Fig 1:**
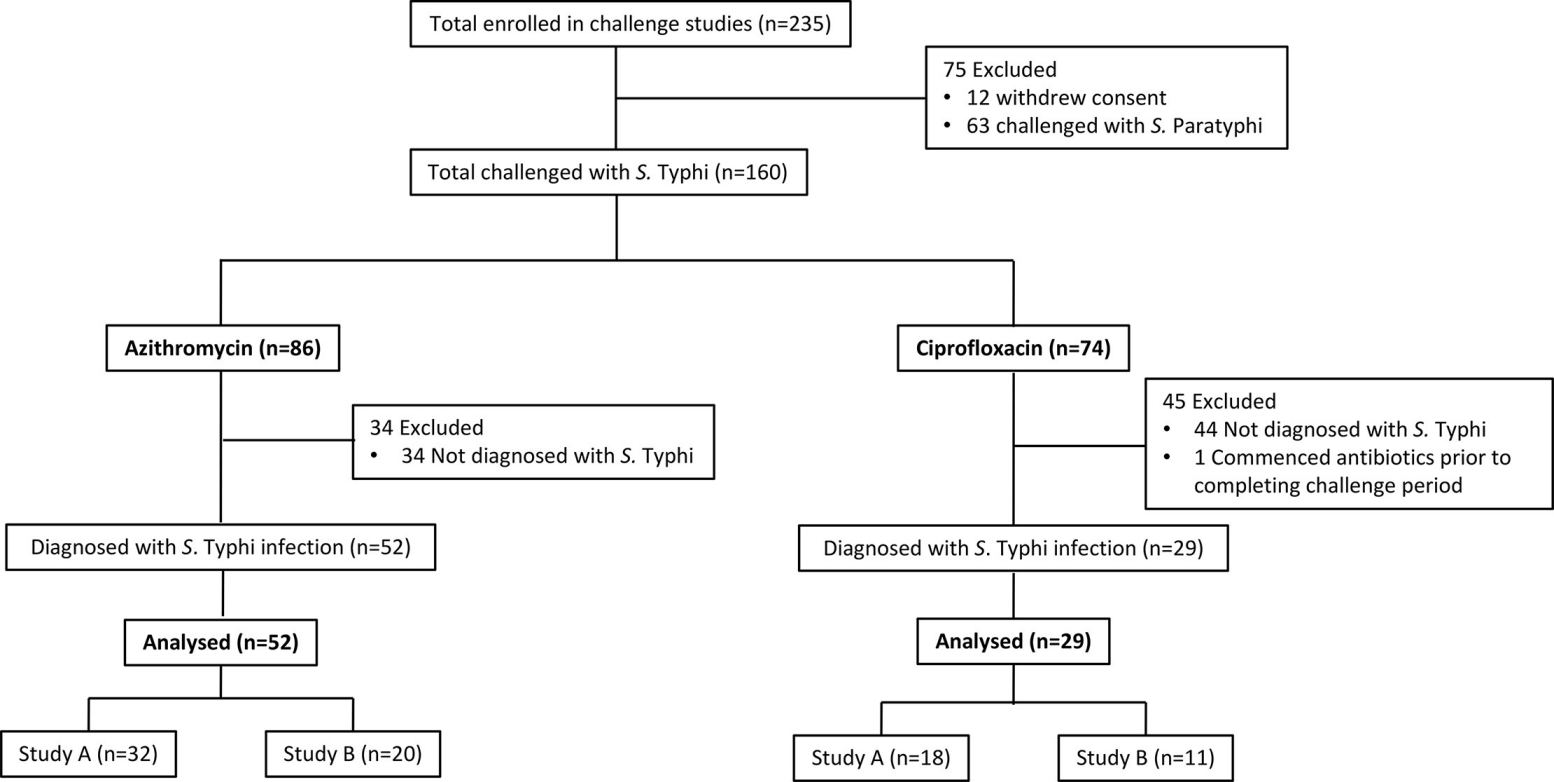
Trial profile for study A and study B.

We enrolled 235 participants between the 17th March 2015 and the 24th August 2017, of whom 160 were challenged with *S*. Typhi. Across both studies, 81/160 challenged participants were diagnosed with typhoid fever–of whom 52 were treated with azithromycin and 29 were treated with ciprofloxacin (Fig 1). Overall, the analysis comprised 62.1% of participants from Study A and 37.9% of participants from Study B (Table 1). Participant baseline characteristics were similar between the azithromycin and ciprofloxacin groups. Of those vaccinated from Study A, 24/50 (48%) had received the control vaccine. In Study B, most participants (19/31; 61%) had no prior exposure to *S*. Typhi (Table 1).

**Table 1:** Baseline participant characteristics. Data are n/N, Control = meningococcal ACWY-CRM conjugate vaccine, Vi-TT = Vi-tetanus toxoid conjugate vaccine, Vi-PS = Vi-polysaccharide vaccine.

	Azithromycin			Ciprofloxacin		
	All (N = 52)	Study A (N = 32)	Study B (N = 20)	All (N = 29)	Study A (N = 18)	Study B (N = 11)
Gender						
Male	34 (65.4%)	20 (62.5%)	14 (70.0%)	23 (79.3%)	14 (77.8%)	9 (81.8%)
Age (Median, IQR)	26.2 [23.3–35.4]	24.4 [22.9–30.7]	27.5 [24.1–44.7]	27.9 [21.6–32.0]	26.6 [21.6–29.6]	27.9 [21.6–40.3]
**Subgroups**						
Vaccine (Study A only)						
Control	16 (30.8%)	16 (50%)	-	8 (27.6%)	8 (44.4%)	-
Vi-TT	7 (13.5%)	7 (21.9%)	-	6 (20.7%)	6 (33.3%)	-
Vi-PS	9 (17.3%)	9 (28.1%)	-	4 (13.8%)	4 (22.2%)	-
None	20 (38.5%)	-	20 (100%)	11 (37.9%)	-	11 (100%)
**Challenge exposure**						
*S*. Typhi naive	37 (71.2%)	32 (100%)	5 (25.0%)	25 (86.2%)	18 (100%)	7 (63.6%)
Previous *S*. Typhi challenge (Study B only)	8 (15.4%)	-	8 (40.0%)	4 (13.8%)	-	4 (36.4%)
Previous *S*. Paratyphi challenge (Study B only)	7 (13.5%)	-	7 (35.0%)	0 (0%)	-	0 (0%)
**Microbiological Outcomes**						
Any *S*. Typhi bacteraemia	52 (100%)	32 (100%)	20 (100%)	26 (89.7%)	16 (88.9%)	10 (90.9%)
*Bacteraemia clearance before commencing antibiotics*	8 (15.4%)	5 (15.6%)	3 (15.0%)	11 (42.3%)	9 (56.3%)	2 (40.0%)
**Clinical Outcomes**						
Any fever ≥38°C	29 (55.8%)	19 (59.4%)	10 (50.0%)	21 (72.4%)	11 (61.1%)	10 (90.9%)
*Fever ≥38°C clearance before commencing antibiotics*	1 (3.4%)	1 (5.3%)	0 (0%)	1 (4.8%)	1 (9.1%)	0 (0%)

**Fig 2:**
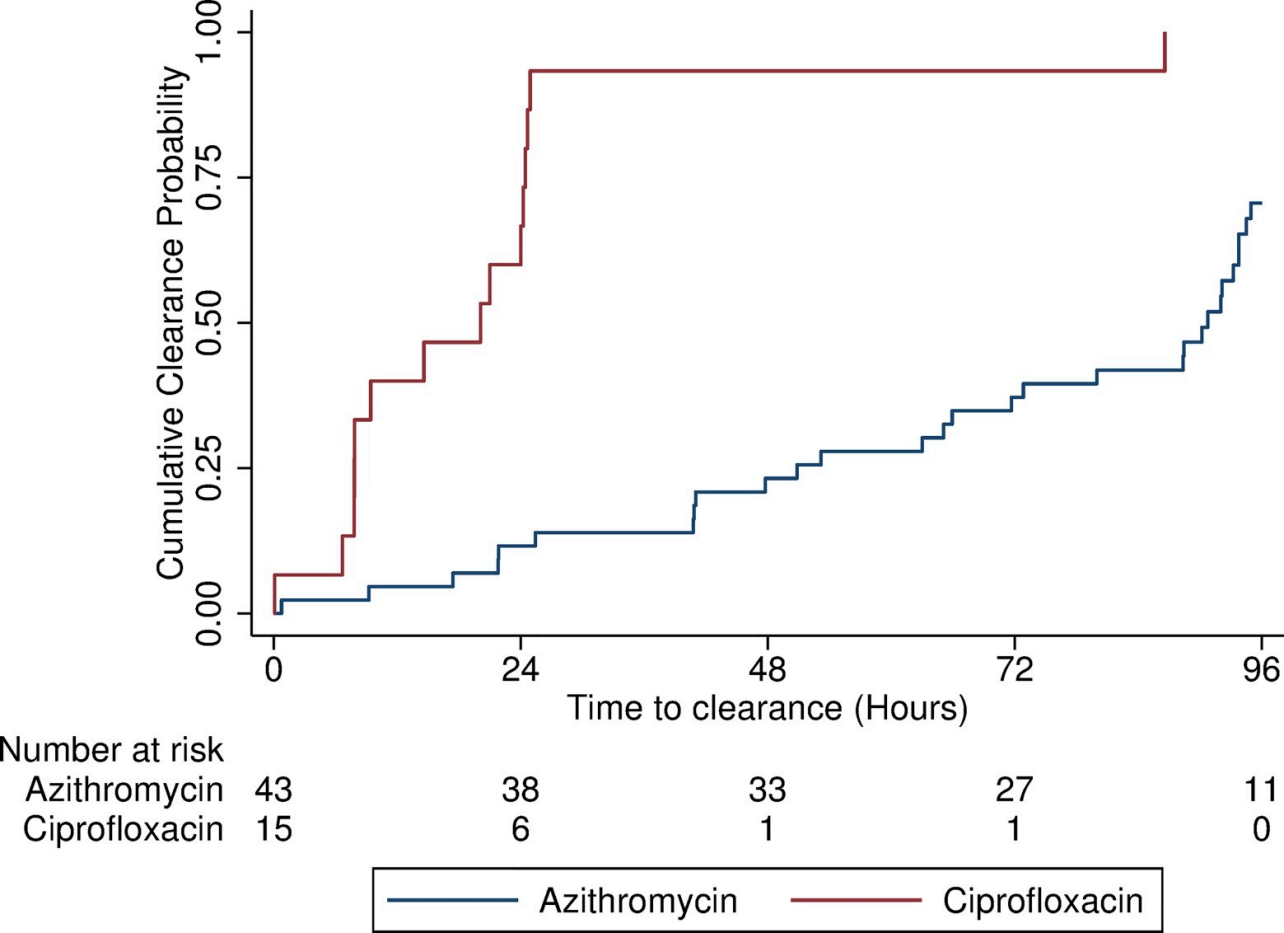
Time to bacteraemia clearance. Log rank test p<0.001.

The duration of *S*. Typhi bacteraemia was significantly prolonged in the azithromycin treated group compared with the ciprofloxacin group (median 90.8 hours [95% CI: 65.9–93.8] vs. 20.1 hours [95% CI: 7.8–24.3], p<0.001—Fig 2). In the unadjusted Cox proportional hazard model, the hazard ratio (HR) for bacteraemia clearance was 9.0 [95% CI: 4.2–19.3] in participants treated with ciprofloxacin compared with those treated with azithromycin. After adjusting for vaccination status, time to antibiotic initiation and prior challenge status, the HR increased to 18.9 [95% CI: 6.6–54.0.3] (Table 2). Of note, adjustment of burden of *S*. Typhi, based on blood quantification results obtained at the time of typhoid diagnosis in Study A, did not affect the association between antibiotic treatment and bacteraemia clearance (S1 Table). Results were consistent when studies were analysed independently and in a sub-group of participants with no history of vaccination or previous challenge (S2 Fig).

**Table 2:** Cox regression analysis for primary and secondary outcomes. * adjusted for study, time to antibiotic commencement, prior challenge status, vaccine status.

	Azithromycin	Ciprofloxacin	p
Primary outcome			
Bacteraemia clearance	N = 43	N = 15	
Crude model	Ref	9.0 (4.2–19.3)	<0.001
Multivariable adjusted*	Ref	18.9 (6.6–54.0)	<0.001
Fever Clearance (Fever ≥38)	N = 27	N = 19	
Crude model	Ref	2.1 (1.1–4.0)	0.03
Multivariable adjusted*	Ref	1.3 (0.6–2.8)	0.51
Secondary outcome			
Stool shedding clearance	N = 19	N = 8	
Crude model	Ref	1.4 (0.6–3.2)	0.48
Multivariable adjusted*	Ref	1.3 (0.4–4.2)	0.68
Fever Clearance (Fever ≥37.5)	N = 33	N = 23	
Crude model	Ref	3.0 (1.6–5.5)	0.001
Multivariable adjusted*	Ref	2.4 (1.2–5.0)	0.02
Any symptoms	N = 50	N = 27	
Crude model	Ref	1.9 (1.2–3.2)	0.01
Multivariable adjusted*	Ref	1.9 (1.1–3.2)	0.03

**Fig 3:**
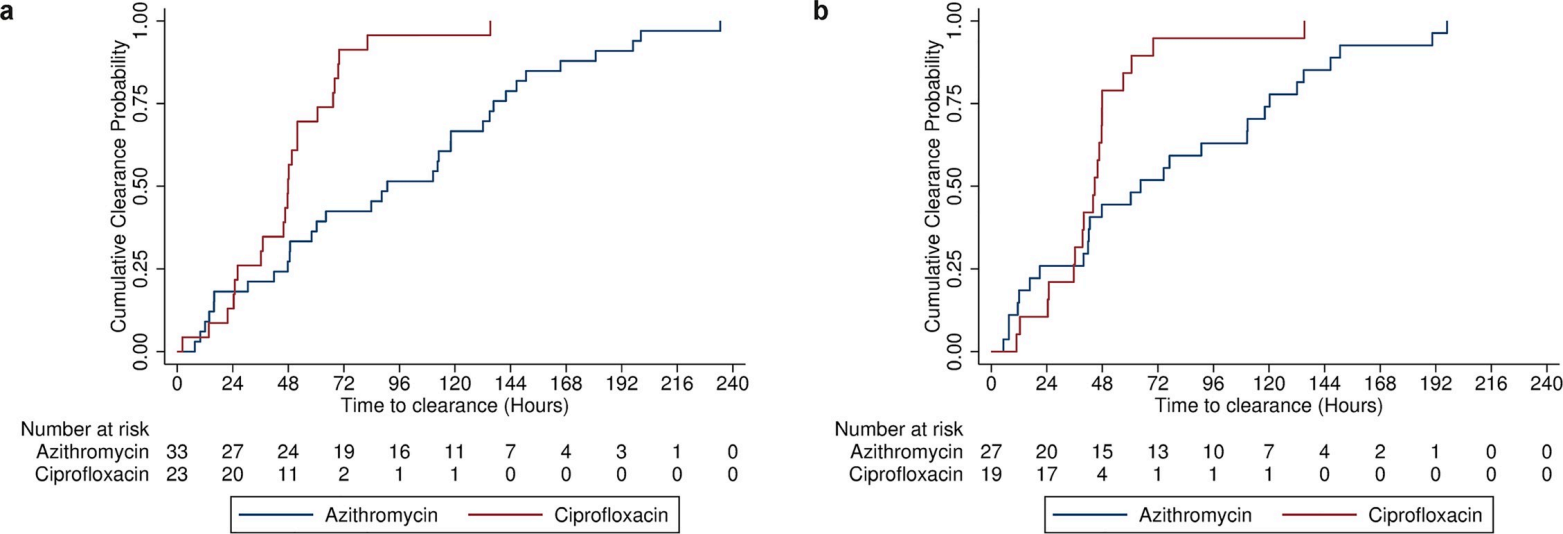
Fever clearance time following initiation of treatment (A) <37.5°C Log rank test p<0.001; (B) <38°C. Unadjusted p value 0.02.

Fever clearance time <38°C was significantly prolonged in azithromycin treated participants (median 65 hours [95% CI: 40–111] vs 45 hours [95% CI: 36–48]; p = 0.02—Fig 3). After adjusting for the pre-specified variables, this association was no longer statistically significant (HR 1.3 [95% CI: 0.6–2.8]; p = 0.51). When using a lower threshold of 37.5°C to define fever, we observed a similar association between FCT and antibiotics in the univariate analysis (log-rank p<0.001, Fig 3). This association remained statistically significant in the multivariable adjusted Cox regression (HR 2.4 [95% CI: 1.2–5.0]; p = 0.02, Table 2). Furthermore, there was a trend towards more rapid symptom resolution, for all solicited symptoms of typhoid fever, in the ciprofloxacin group than the azithromycin treated group, log-rank p = 0.006 (S1 Fig).

More participants in the azithromycin treated group (28/52, 53.8%) had prolonged treatment responses than the ciprofloxacin group (1/29, 3.4%). Twelve azithromycin-treated participants (23.1%) had bacteraemia detected ≥72 hours after commencing azithromycin and fever ≥38°C (Table 3). Consequently, more azithromycin treated participants had their antibiotic treatment changed to ciprofloxacin (14/52, 26.9%). Rates of liver enzyme elevation in Study A participants were similar between the two treatment groups. Of note, a small proportion of participants met the definition for drug-induced liver injury based on raised ALT levels (azithromycin 6.3%, ciprofloxacin 5.6%; Table 3).

**Table 3:** Secondary outcomes.

	Azithromycin			Ciprofloxacin			
	All (N = 52)	Study A (N = 32)	Study B (N = 20)	All (N = 29)	Study A (N = 18)	Study B (N = 11)	P value*
**Prolonged Treatment Response**							
Total number of participants with prolonged treatment responses	28 (53.8%)	18 (56.3%)	10 (50.0%)	1 (3.4%)	0 (0%)	1 (9.1%)	<0.001
Positive blood culture with *S*. Typhi for ≥72 hours after commencing antibiotics	27 (51.9%)	18 (56.3%)	9 (45.0%)	1 (3.4%)	0 (0%)	1 (9.1%)	<0.001
Persistent fever ≥38°C for ≥72 hours	13 (25.0%)	8 (25.0%)	5 (25.0%)	1 (3.4%)	0 (0%)	1 (9.1%)	0.02
Both positive blood culture and persistent fever ≥38°C for ≥72 hours	12 (23.1%)	8 (25.0%)	4 (20.0%)	1 (3.4%)	0 (0%)	1 (9.1%)	0.03
**Treatment Change**							
Antibiotic treatment change in diagnosed participants	14 (26.9%)	11 (34.4%)	3 (15.0%)	2 (6.9%)	1 (5.6%)	1 (9.1%)	0.04
Median time to treatment change (diagnosed participants) in days (Median, IQR)	4 [3–4]	4 [3–4]	4 [3–6]	11.5 [9–14]	9 [9–9]	14 [14–14]	<0.01
Antibiotic change due to adverse reaction or clinical concern (not meeting above definitions)	6 (11.5%)	3 (9.4%)	3 (15.0%)	1 (3.4%)	1 (5.6%)	0 (0%)	0.41
**Liver Enzyme Derangement (Study A only)**							
Any increase in Liver Enzymes	-	8 (25.0%)	-	-	4 (22.2%)	-	1.0
ALT ≥1.1x ULN	-	5 (15.6%)	-	-	2 (11.1%)	-	1.0
ALP ≥1.1x ULN	-	1 (3.1%)	-	-	1 (5.6%)	-	1.0
Bilirubin ≥1.1x ULN	-	2 (6.3%)	-	-	1 (5.6%)	-	1.0
Drug-induced liver injury (enzyme derangement meeting DILI criteria)	-	2 (6.3%)	-	-	1 (5.6%)	-	1.0
ALT ≥5x ULN	-	2 (6.3%)	-	-	1 (5.6%)	-	1.0
ALP ≥2x ULN	-	0 (0%)	-	-	0 (0%)	-	1.0
ALT ≥3x ULN and Bilirubin ≥2x ULN	-	0 (0%)	-	-	0 (0%)	-	1.0

* p-value for the overall comparison.

**Fig 4:**
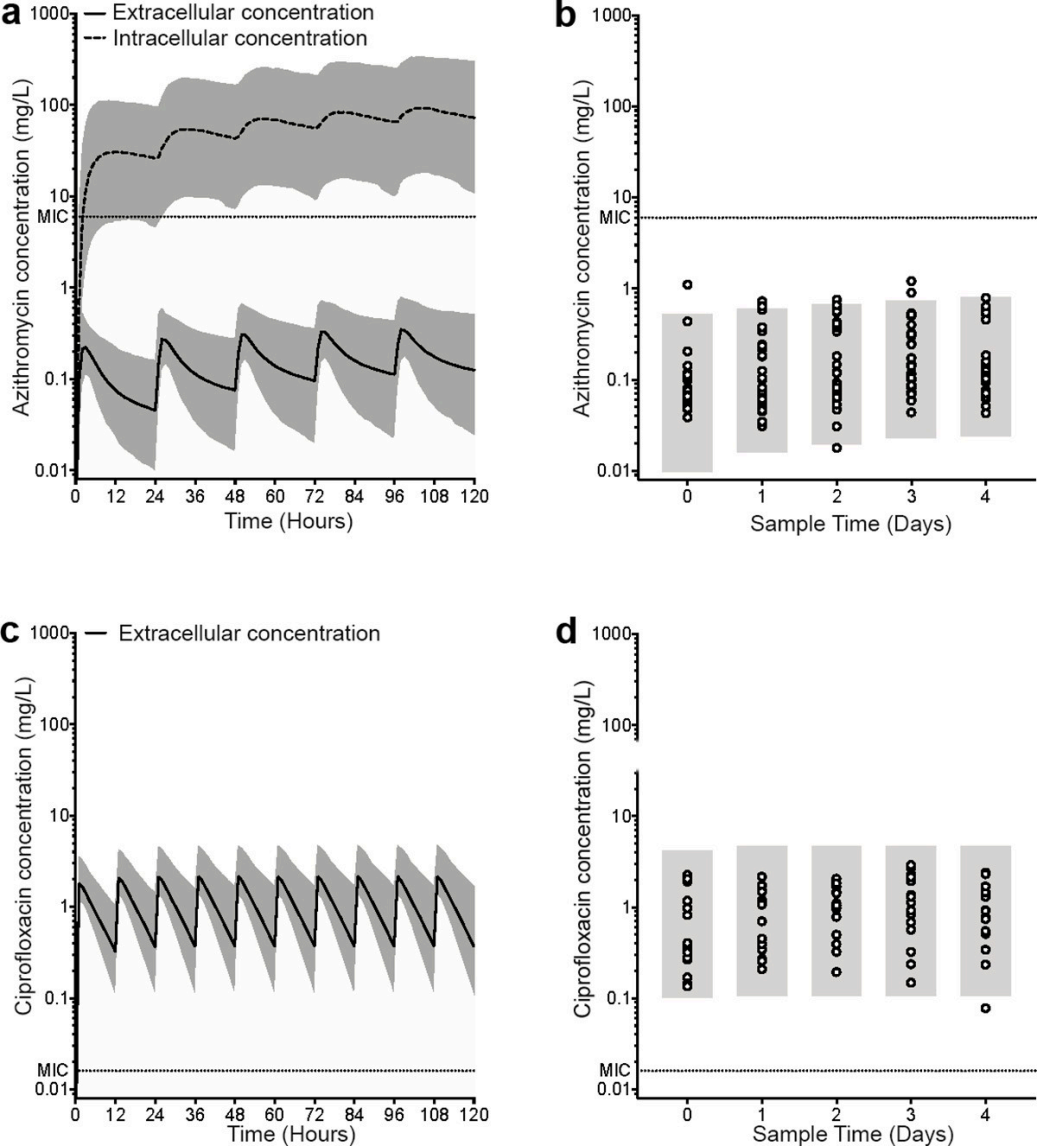
PK simulations and observed plasma concentrations. (A and C) PK simulations showing (A) azithromycin 500 mg daily and (C) ciprofloxacin 500 mg twice daily. The solid black line represents the median predicted plasma concentration. The dotted black line represents the median predicted intracellular concentration. The grey area represents the 5^th^-95^th^ percentile. The horizontal dotted line represents the minimum inhibitory concentration (MIC) (B and D) Open circles represent observed plasma concentrations. The grey area represents the minimum 5^th^ and maximum 95^th^ percentile for each day as predicted in A and C. The horizontal dotted line represents the MIC.

For both azithromycin and ciprofloxacin, serum concentrations in samples collected each day during treatment were consistent with published PK data [33,34]. Of note, plasma concentrations of azithromycin did not exceed the MIC at any time point (Fig 4B). PK simulations however, demonstrated intracellular accumulation of azithromycin exceeding the MIC over the treatment period (Fig 4A). Further PK simulations demonstrated that administration of a 1,000mg loading dose followed by 500mg daily dosing of azithromycin would not increase extracellular levels beyond the MIC but would likely result in more patients achieving intracellular concentrations in excess of the MIC within the first 24 hours of treatment (S3 Fig). Additional PK simulations of alternative dosing regimens, including 1,000mg daily dosing of azithromycin and 500mg twice daily dosing of azithromycin, demonstrated similar findings (rapid accumulation of intracellular azithromycin with extracellular concentrations below the MIC, S4 Fig). Concentrations of ciprofloxacin in serum exceeded the MIC at all time points (Fig 4D).

## Discussion

The emergence and dissemination of multidrug resistant, fluoroquinolone resistant and extensively drug resistant (XDR) strains of *S*. Typhi represents a threat to the global control of enteric fever[9]. Azithromycin is one of the few oral treatment options to which the majority of global *S*. Typhi isolates remain sensitive. In this study, we performed an analysis of treatment responses to ciprofloxacin and azithromycin following challenge with a fully sensitive strain of *S*. Typhi in over 100 healthy volunteers. All patients were successfully treated. However, when a diverse range of objective clinical and microbiological endpoints were used to assess treatment responses, we observed a delayed response to treatment of *S*. Typhi infection in those receiving azithromycin compared with those receiving ciprofloxacin.

The median *S*. Typhi fever clearance time and duration of bacteraemia reported in our study is comparable to that reported in field studies. Azithromycin has been associated with fever clearance times of ≥96 hours [13,15,16,18,19] and prolonged bacteraemia [18,19] in several field trials–however not to the extent to which was observed in this study. It is possible that this discrepancy is a result of the high inoculum dose used in our CHI model in a relatively-immunologically naïve cohort compared with endemic settings. Therefore, while comparisons made directly to ciprofloxacin are valid, data concerning the limited efficacy of azithromycin are not entirely reflective of clinical observations in the field.

In some studies, prolonged bacteraemia has been associated with clinical deterioration and treatment failure [22] supporting the argument for rapid bacterial clearance to reduce fever clearance time and improve clinical recovery. Conversely, prolonged fever clearance and bacteraemia may sit on the spectrum of normal responses to azithromycin treatment. An apparent failure to improve may prompt clinicians to escalate or switch therapy, interrupting and ultimately prolonging treatment courses. Patients should be adequately counselled regarding likely recovery time to prevent potential non-compliance with treatment.

The optimal dosing regimen of azithromycin for the treatment of enteric fever is yet to be determined. Most studies use a regimen of 10-20mg/kg/day for 5 to 7 days [35]. Some trials have used a loading dose of 1,000mg azithromycin at day 1 followed by 6 days of treatment with 500mg/day. This regimen has not been associated with significant differences in fever clearance time when compared with fluoroquinolone treatment [14,15]. Azithromycin has excellent tissue penetration, particularly of white blood cells, which often harbour intracellular *S*. Typhi bacteria [36]. However, it is possible that extracellular bacteria are not adequately controlled due to the low peak serum concentration achieved relative to the MICs of susceptible strains (0.4mg/L after a single 500mg dose) [37–39]. In this study, we observed that extracellular concentrations of azithromycin did not exceed the MIC. Given that azithromycin accumulates approximately 200-fold within the intracellular space of macrophages [12], concentrations within the intracellular space are likely to be in excess of the MIC. PK simulations carried out here, suggest that a 1,000mg loading dose of azithromycin would not sufficiently increase extracellular concentrations beyond the MIC and would, therefore, not result in a significant increase in the elimination rate of extracellular *S*. Typhi. However, on account of the intracellular accumulation of azithromycin, a 1,000mg loading dose is predicted to result in more patients achieving intracellular concentrations in excess of the MIC within the first 24 hours of treatment. As such, a loading dose of azithromycin may help to control the overall burden of bacteria, improving bacterial clearance and treatment response.

The response to ciprofloxacin treatment was comparable to that observed in field trials with fully susceptible strains [40]. Strains of *S*. Typhi with an MIC ≤0.03 –such as the strain used in this study—display an excellent response to fluoroquinolones allowing for short-course therapy [41] [42]. The PK/PD parameter that predicts efficacy is peak MIC or AUC/MIC. We have demonstrated that during treatment with ciprofloxacin at 500mg twice daily, intra- and extracellular concentrations are consistently above the MIC. Given that ciprofloxacin is also known to accumulate approximately 5-fold within the intracellular space of macrophages [43], 500mg twice daily dosing of ciprofloxacin will result in intracellular concentrations in excess of the MIC. Therefore, while azithromycin would appear to only target intracellular populations when used at standard clinical doses, ciprofloxacin may simultaneously target both intracellular and extracellular populations. In acute typhoid fever, approximately 40% of circulating *S*. Typhi bacilli are thought to reside in the extracellular compartment [36]. These observations lead us to hypothesise that the low systemic plasma concentrations of azithromycin result in little to no elimination of extracellular bacteria resulting in an extended duration of bacteraemia and fever clearance time relative to ciprofloxacin.

Resistance to azithromycin amongst circulating strains is currently thought to be uncommon, although the requirement to confirm by MIC makes reliable data scarce. Azithromycin resistance is known to be mediated via mutations in the *ereA*, *msrD* and *msrA* genes [4]–as well as a recently described mutation in a gene encoding an efflux pump *acrB*[44]*—*which are not present in the *S*. Typhi strain used in the CHI model [45]. Resistance rates are likely to rise with increasing use of these drugs–including in mass-drug administration campaigns [46,47]. Robust national and regional surveillance systems to monitor trends in antibiotic resistance are required to ensure that treatment guidelines provide relevant recommendations, however quality assured diagnostic microbiology facilities to inform surveillance remain scarce in many low-income countries.

Combined interventions including vaccination; provision and access to safe water; hygiene interventions and improvements to sanitation infrastructure are required if global control of enteric fever is to be achieved. In October 2017, the WHO Strategic Advisory Group of Experts (SAGE) on immunisation recommended programmatic use of TCVs in children over six months of age in typhoid endemic countries [48]. Programmatic use of TCVs is anticipated to reduce overall antibiotic consumption in highly endemic areas, by preventing both confirmed typhoid cases requiring antibiotic treatment, and by reducing the incidence of undifferentiated fever treated with undirected therapy. Immunisation forms a central pillar of the global action plan on antimicrobial resistance and the deployment of TCVs, in line with WHO recommendations, could have a major impact on the burden of typhoid fever and on the spread of antibiotic resistance in typhoidal *Salmonella* [49].

We acknowledge the limitations of our experimental approach. Human challenge studies are usually performed with well characterised, fully antibiotic-sensitive challenge strains to ensure that a broad range of therapeutic options are available to treat participants. Such strains may not be representative of contemporary circulating strains of *S*. Typhi, such as the MDR-associated H58 (genotype 4.3.1) strain of *S*. Typhi [4]. Strain-specific differences in treatment response could be studied by developing CHI studies with a diverse range of *S*. Typhi challenge stocks, including antibiotic sensitive H58 (4.3.1) strains. Several recent studies suggest that fluoroquinolones should not be used for the empirical treatment of enteric fever in South/South East Asia, due to fluoroquinolone-resistance resulting in treatment failures [6]. In addition, the European Medicines Agency has raised several safety concerns related to fluoroquinolone use, with specific reference to disabling and potentially permanent adverse events [50]. While our results may not be directly applicable to treatment options in endemic settings where fluoroquinolone-resistance is prevalent, we believe there are several relevant observations from this study that should be considered when managing uncomplicated enteric fever in adults.

Another limitation of the study was the absence of randomisation of antibiotic allocation for Study B. In Study A, randomisation took place at the time of enrolment, but not after typhoid diagnosis. This may have resulted in an imbalance of sample size and potentially covariates. However, after adjusting for the key covariates, pre-specified in the statistical analysis plan, most of the results were consistent with the unadjusted analysis.

In summary, oral azithromycin is an effective outpatient treatment option for adults with uncomplicated enteric fever and should be used in high-burden countries where fluoroquinolone-resistance is common. Administration of a loading dose of azithromycin should be considered, as it will assist with increasing intracellular concentrations beyond the MIC in more patients during the first 24 hours of treatment. In the era of increasing fluoroquinolone-resistance and apparent re-emergence of sensitivity to traditional first line agents [51], further studies are required to assess novel treatment strategies, including appropriate azithromycin dosing regimens; novel antimicrobials; antibiotic cycling; combination therapies and treatment options in children. Pending the assessment of new treatment strategies, we advocate for the deployment of TCVs in line with WHO recommendations, to improve child health and limit the spread of antibiotic resistant *S*. Typhi.

## Supporting information

S1 Table Cox regression analysis for primary and secondary outcomes per study. * adjusted for time to antibiotic commencement, prior challenge status, vaccine status; ^§^ further adjusted for burden of S. Typhi in Study A as measured using quantitative culture.
(DOCX)

S1 Fig Time to symptom resolution. Log rank test: p = 0.006.
(DOCX)

S2 Fig Kaplan-Meir curves illustrating time to blood culture clearance in study sub-groups, (a) Study A; (b) Study B; (c) Participants with no prior vaccine history (Study A only); (d) Participants with no history of previous typhoid challenge.
(DOCX)

S3 Fig PK simulation showing 1,000 mg loading dose followed by 500 mg daily azithromycin. The solid black line represents the median predicted plasma concentration. The dotted black line represents the median predicted intracellular concentration. The grey area represents the 5th-95th percentile. The horizontal dotted line represents the minimum inhibitory concentration (MIC).
(DOCX)

S4 Fig a) PK simulation showing 1,000mg daily dosing of azithromycin; b) PK simulation showing 500mg twice daily dosing of azithromycin. The solid black line represents the median predicted plasma concentration. The dotted black line represents the median predicted intracellular concentration. The grey area represents the 5th-95th percentile. The horizontal dotted line represents the minimum inhibitory concentration (MIC).
(DOCX)

S1 Data (CSV)
