# Supplementary material for: Treatment responses to Azithromycin and Ciprofloxacin in uncomplicated Salmonella Typhi infection: A comparison of Clinical and Microbiological Data from a Controlled Human Infection Model
Source: PLoS Negl Trop Dis. 2019 Dec 26;13(12):e0007955. doi: 10.1371/journal.pntd.0007955 (PMC6948818; doi:10.1371/journal.pntd.0007955)
Supplement: S1 Table — * adjusted for time to antibiotic commencement, prior challenge status, vaccine status; § further adjusted for burden of S. Typhi in Study A as measured using quantitative culture. (DOCX) [file pntd.0007955.s001.docx]

S1 Table - Cox regression analysis for primary and secondary outcomes per study.* adjusted for time to antibiotic commencement, prior challenge status, vaccine status; ^§^ further adjusted for burden of S. Typhi in Study A as measured using quantitative culture

|  | Study A | | | Study B | | |
| --- | --- | --- | --- | --- | --- | --- |
|  | Azithromycin | Ciprofloxacin | p | Azithromycin | Ciprofloxacin | p |
| Primary outcome |  |  |  |  |  |  |
| Bacteraemia clearance | N=26 | N=7 |  | N=17 | N=8 |  |
| Crude model | Ref | 61.3  (7.2-523.6) | <0.001 | Ref | 4.6  (1.7-13.0) | 0.003 |
| Multivariable adjusted^*^ | Ref | 81.3  (8.2-805.0) | <0.001 | Ref | 12.1  (2.4-61.4) | 0.003 |
| Multivariable adjusted^§^ | Ref | 49.0  (5.2-459.1) | 0.001 |  |  |  |
| Fever Clearance (Fever ≥38) | N=18 | N=10 |  | N=9 | N=9 |  |
| Crude model | Ref | 2.4 (1.0-5.9) | 0.06 | Ref | 1.8 (0.7-4.7) | 0.26 |
| Multivariable adjusted^*^ | Ref | 1.2 (0.5-3.2) | 0.68 | Ref | 1.6 (0.4-5.8) | 0.49 |
| Secondary outcome |  |  |  |  |  |  |
| Stool shedding clearance | N=10 | N=6 |  | N=9 | N=2 |  |
| Crude model | Ref | 1.1 (0.4-3.2) | 0.82 | Ref | 4.6 (0.6-33.0) | 0.13 |
| Multivariable adjusted^*^ | Ref | 1.0 (0.3-3.5) | 0.98 | Ref | 5.4 (0.3-101.0) | 0.25 |
| Fever Clearance (Fever ≥37.5) | N=20 | N=13 |  | N=13 | N=10 |  |
| Crude model | Ref | 4.1 (1.7-10.2) | 0.002 | Ref | 2.2 (0.9-5.7) | 0.09 |
| Multivariable adjusted^*^ | Ref | 2.5 (0.9-7.0) | 0.07 | Ref | 2.3 (0.7-7.5) | 0.16 |
| Any symptoms | N=31 | N=17 |  | N=19 | N=10 |  |
| Crude model | Ref | 1. 5 (0.8-2.9) | 0.21 | Ref | 2.9 (1.2-6.7) | 0.02 |
| Multivariable adjusted^*^ | Ref | 1.4 (0.7-2.8) | 0.28 | Ref | 3.7 (1.2-11.1) | 0.02 |
