# Supplementary figures and images for: Treatment responses to Azithromycin and Ciprofloxacin in uncomplicated Salmonella Typhi infection: A comparison of Clinical and Microbiological Data from a Controlled Human Infection Model
Source: PLoS Negl Trop Dis. 2019 Dec 26;13(12):e0007955. doi: 10.1371/journal.pntd.0007955 (PMC6948818; doi:10.1371/journal.pntd.0007955)

S1 Figure – Time to symptom resolution. Log rank test: p=0.006


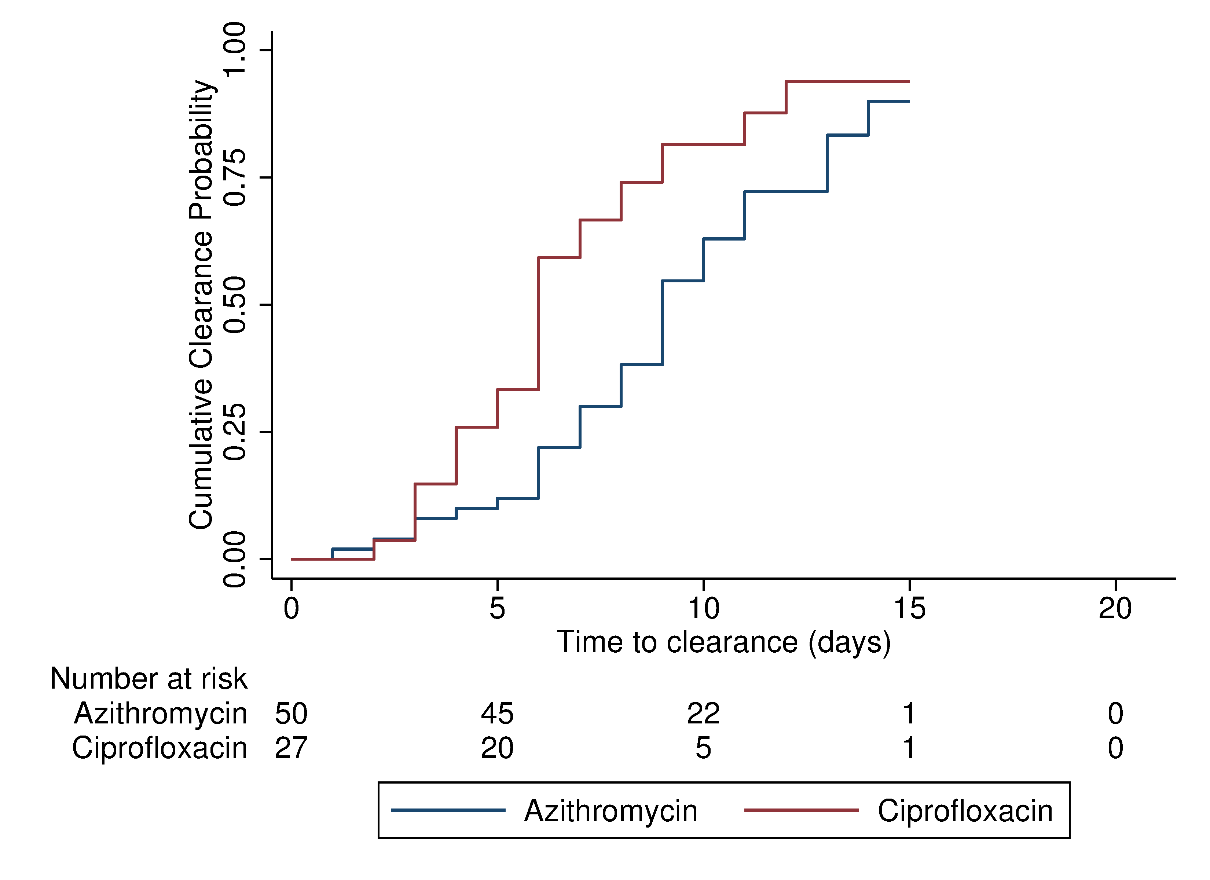

Supplement: S1 Fig — Log rank test: p = 0.006. (DOCX) [file pntd.0007955.s002.docx]
