# Supplementary material for: Treatment responses to Azithromycin and Ciprofloxacin in uncomplicated Salmonella Typhi infection: A comparison of Clinical and Microbiological Data from a Controlled Human Infection Model
Source: PLoS Negl Trop Dis. 2019 Dec 26;13(12):e0007955. doi: 10.1371/journal.pntd.0007955 (PMC6948818; doi:10.1371/journal.pntd.0007955)

S2 Figure – Kaplan-Meir curves illustrating time to blood culture clearance in study sub-groups, (a) Study A; (b) Study B; (c) Participants with no prior vaccine history (Study A only); (d) Participants with no history of previous typhoid challenge.


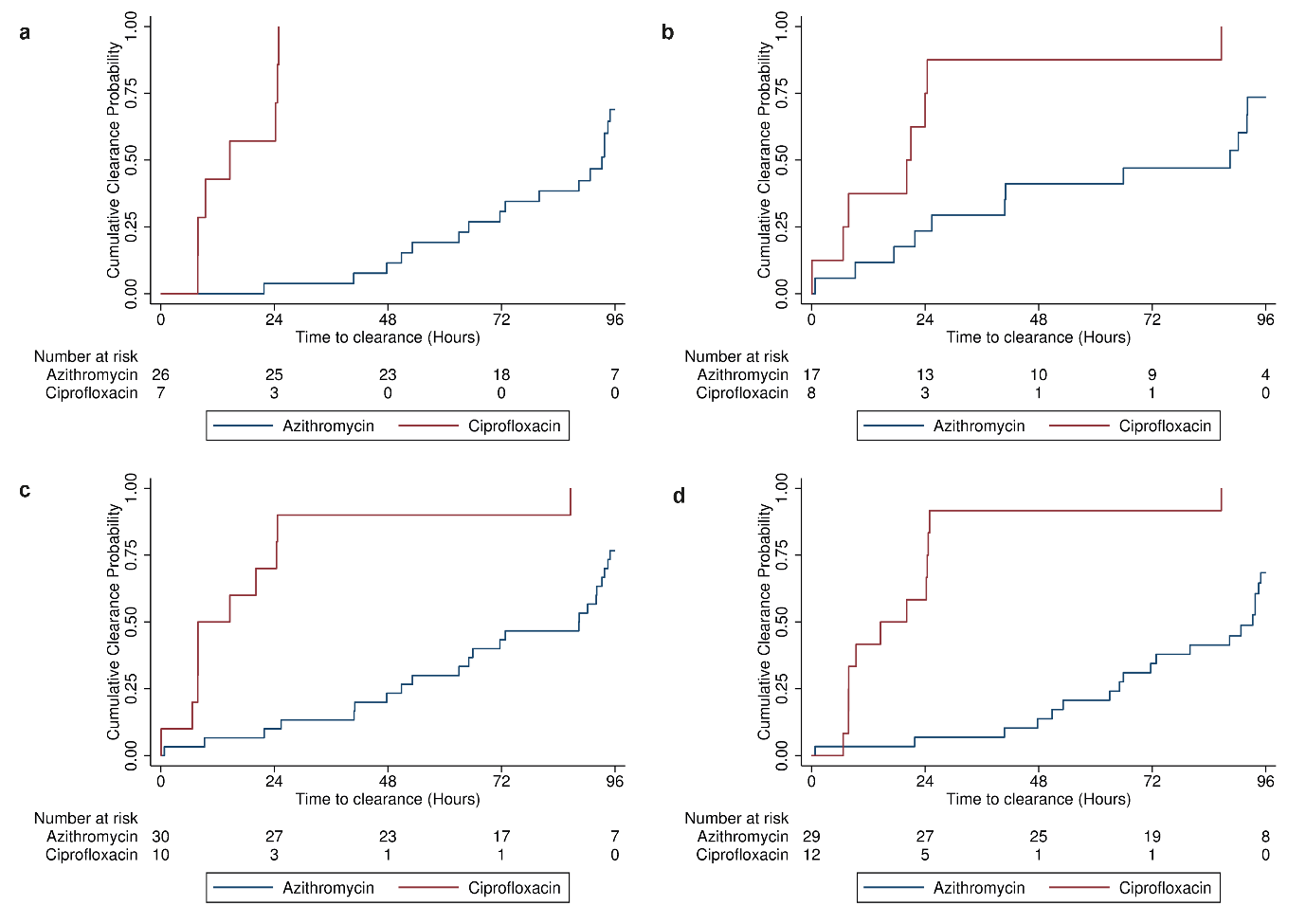

Supplement: S2 Fig — Kaplan-Meir curves illustrating time to blood culture clearance in study sub-groups, (a) Study A; (b) Study B; (c) Participants with no prior vaccine history (Study A only); (d) Participants with no history of previous typhoid challenge. (DOCX) [file pntd.0007955.s003.docx]
