# Supplementary material for: Treatment responses to Azithromycin and Ciprofloxacin in uncomplicated Salmonella Typhi infection: A comparison of Clinical and Microbiological Data from a Controlled Human Infection Model
Source: PLoS Negl Trop Dis. 2019 Dec 26;13(12):e0007955. doi: 10.1371/journal.pntd.0007955 (PMC6948818; doi:10.1371/journal.pntd.0007955)

S4 Figure – a) PK simulation showing 1,000mg daily dosing of azithromycin; b) PK simulation showing 500mg twice daily dosing of azithromycin. The solid black line represents the median predicted plasma concentration. The dotted black line represents the median predicted intracellular concentration. The grey area represents the 5th-95th percentile. The horizontal dotted line represents the minimum inhibitory concentration (MIC).


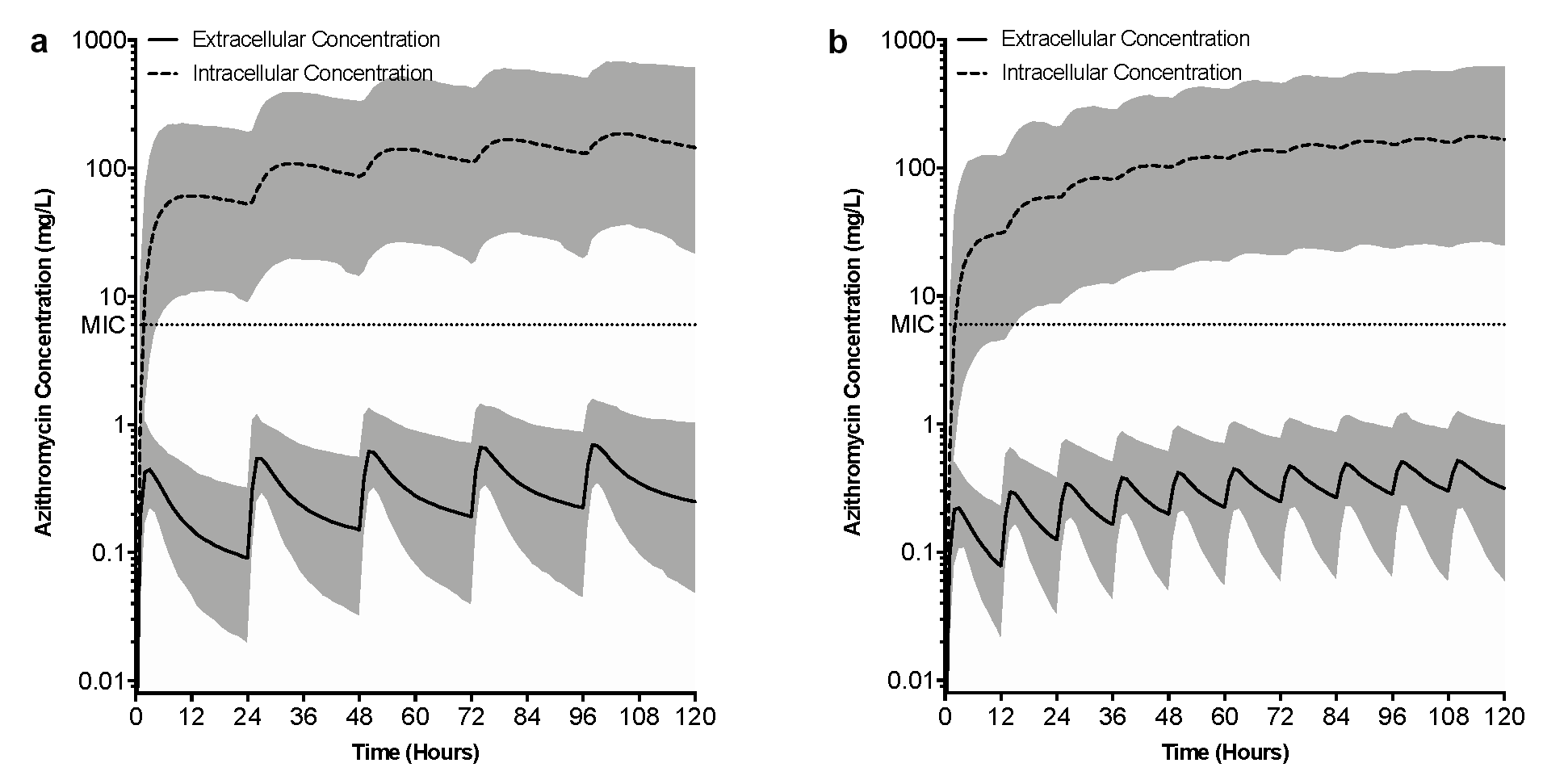

Supplement: S4 Fig — a) PK simulation showing 1,000mg daily dosing of azithromycin; b) PK simulation showing 500mg twice daily dosing of azithromycin. The solid black line represents the median predicted plasma concentration. The dotted black line represents the median predicted intracellular concentration. The grey area represents the 5th-95th percentile. The horizontal dotted line represents the minimum inhibitory concentration (MIC). (DOCX) [file pntd.0007955.s005.docx]
